# Host Factors Genes *BcCLC1* and *BcCLC2* Confer Turnip Mosaic Virus Resistance in Non-Heading Chinese Cabbage (*Brassica campestris* ssp. *chinensis*)

**DOI:** 10.3390/plants12122269

**Published:** 2023-06-10

**Authors:** Mengguo Yuan, Shanwu Lyu, Yaolong Wang, Liu E, Tongkun Liu, Xilin Hou, Ying Li, Changwei Zhang

**Affiliations:** 1 National Key Laboratory of Crop Genetics & Germplasm Enhancement and Utilization, Nanjing Agricultural University, Nanjing 210095, China; 2 College of Horticulture, Nanjing Agricultural University, Nanjing 210095, China; 3 Key Laboratory of South China Agricultural Plant Molecular Analysis and Genetic Improvement, Guangdong Provincial Key Laboratory of Applied Botany, South China Botanical Garden, Chinese Academy of Sciences, Guangzhou 510650, China

**Keywords:** clathrin, TuMV, *BcCLCs*, non-heading Chinese cabbage, protein interaction

## Abstract

Clathrin is an evolutionarily highly conserved evolutionary protein consisting of clathrin light chains (CLC) and clathrin heavy chains (CHC), and these form its basic structure. Clathrin is an important host factor in the process of viral infection. In this study, we cloned the *BcCLC1* gene and the *BcCLC2* gene from the ‘49CX’ variety of non-heading Chinese cabbage (NHCC, *Brassica campestris* L. ssp. *chinensis* Makino) and verified their functions. The results showed that *BcCLC1* was mainly localized in the cytomembrane and cytoplasm, and only a small amount entered the nucleus. *BcCLC2* encoded a protein comprising 265 amino acids that were distributed in the cytomembrane, nucleus, and cytoplasm. A BiFC assay and yeast two-hybrid (Y2H) analysis showed that *BcCLCs* (*BcCLC1* and *BcCLC2*) could interact with several TuMV proteins. We further investigated the mechanism of *BcCLCs* in regulating TuMV virus infections in NHCC, and observed that *BcCLCs* gene silencing inhibited TuMV infections and overexpression of *BcCLCs* in *Arabidopsis* promoted TuMV infections in NHCC. Finally, mutants of *Arabidopsis* homologs of *BcCLCs* were also screened and subjected to TuMV inoculation tests. In conclusion, we speculate that *BcCLCs* confer *Turnip mosaic virus* (TuMV) resistance in NHCC by interacting with TuMV proteins to promote the intracellular transport of the virus.

## 1. Introduction

Clathrin plays an important role in mediating the viral invasion of host cells, and has been most intensively studied in the process of cell infection caused by animal viruses. Although some animal viruses can enter the cytoplasm directly through their penetration ability, most require the assistance of endocytosis. Cells internalize and respond to viruses through multiple endocytic pathways. Early studies have revealed that adenovirus 2,5 and vesicular stomatitis virus (VSV) accumulate in a thickened region of the cell membrane, a region where clathrin-mediated endocytic (CME) vesicles are produced [1,2]. The first virus that was found to explore the CME efficient infection pathway was the Semliki Forest virus (SFV) [3]. Then we started finding many other viruses, such as *Sindbis virus* (SINV) [4,5], dengue virus (DENV), etc. [6,7]. Initially, it was thought that the formation of clathrin-encapsulated vesicles, as a basic biological process, occurred in all cases. In recent years, it has been found that the formation of vesicles is facilitated by cargo loading in vesicles, and viruses, such as VSV and influenza A viruses, can promote the production of vesicles wrapped by clathrin [8,9,10]. In the CME pathway, other proteins besides clathrin, such as dynamin and AP2, are likewise involved in the infection process of the virus [9,10]. There are also several reports of endocytosis pathway-mediated pathogen infections in plants. If we inhibit the expression of *Pl3K*, we can reduce endocytosis and ROS production in cells and improve disease resistance in *Medicago* L. [11]. As mentioned previously, the *Arabidopsis* synaptotagmin (SYTA) is localized in endosomes and interacts with the intercellular movement proteins of *Cabbage leaf curl virus* (CaLCuV) and *Tobacco mosaic virus* (TMV). In SYTA knockout mutants, the intercellular movement of the virus was inhibited, demonstrating that SYTA regulates endocytosis and the transport of its cargo by viral movement proteins to the plasmodesmata for intercellular dissemination via the endocytic recycling pathway [12]. This, as well as the discovery of the role played by dynamin and AP2β in the CME pathway’s involvement in infection and promotion of viral replication, were discovered by Wu et al. [13,14]. As a side note, the clathrin proteins of NHCC likely have similar functions, with viruses recruiting clathrin, accelerating intracellular vesicular transport, and facilitating processes such as virus replication and intercellular transport.

Plant clathrin is an evolutionarily highly conserved protein with a triskelion as its basic structural unit. Each triskelion backbone contains three clathrin light chains (CLC) and three clathrin heavy chains (CHC) proteins [15]. The loss of CLC2 and CLC3 functions in *Arabidopsis* alters the endocytosis, intracellular transport, and signal transduction of the growth hormone’s export polarity transporter PIN(PIN-FORMED) localized in the plasma membrane, which in turn leads to various intracellular phenotypic alterations [16]. The results, such as shortened roots, elongated hypocotyls, and blunted root grounding responses, all of which are similar to growth hormone-related phenotypic defects [17,18,19,20], suggest that endocytosis mediated by lattice proteins plays a role in growth hormone polarity transportation and signal transduction. The wheat light chain gene *TaCLC1* is involved in Fusarium head blight(FHB) resistance of wheat by promoting the expression of PR proteins, and is resistant to FHB in wheat, *Nicotiana benthamiana*, and *Arabidopsis* [21].

During the growth of NHCC, yields could be reduced by the *Turnip mosaic virus* (TuMV), which is the main disease affecting the quality and yield of NHCC. The absence of clathrin causes defects in development, cytoplasmic division, and osmoregulation in plants and animals [22], but the mechanism of interaction between clathrin proteins and TuMV in NHCC has not been investigated. Here, the clathrin light proteins were selected for functional verification. NHCC clathrin genes show a close homology with *Arabidopsis*. *BcCLC1* and *BcCLC2* overexpressing and silencing plants showed opposite trends through quantitative real-time PCR (qRT-PCR). In verifying gene function, we found that *BcCLC1* and *BcCLC2* silenced plants inhibited by TuMV infection but the overexpression of *BcCLC1* and *BcCLC2* promoted TuMV to infect NHCC. Following the interaction verifications with a bimolecular fluorescent complimentary (BiFC) assay and yeast two-hybrid (Y2H) analysis, we found that many TuMV proteins can interact with *BcCLCs*, such as VPg, 6K2, and CP. This suggested that the *BcCLCs* are instrumental in TuMV infection. These results have some reference values for NHCC in TuMV resistance.

## 2. Results

### 2.1. TuMV Infection Increased the Expression Level of BcCLCs

**Figure 1:**
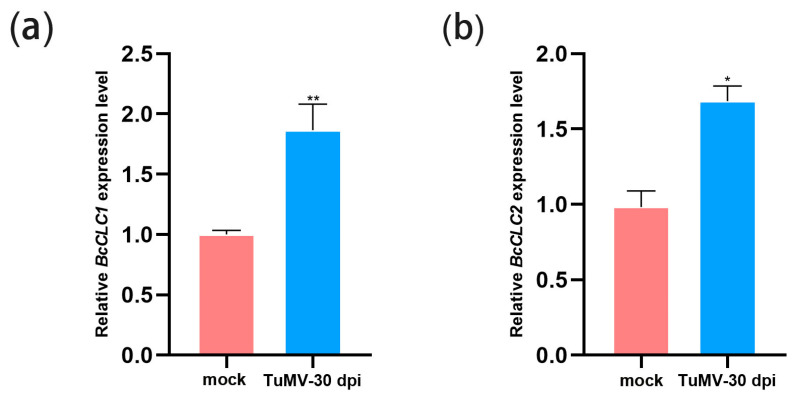
TuMV infection increased the expression level of *BcCLCs*. (**a**) RT-qPCR analysis of *BcCLC1* relative expression level in mock (infiltrated with buffer) and TuMV-infected NHCC; (**b**) RT-qPCR analysis of *BcCLC2* relative expression level in mock (infiltrated with buffer) and TuMV-infected NHCC; Differences between data were detected by Student’s *t*-test, *, *p* < 0.05, **, *p* < 0.01.

To define the effect of TuMV infection on *BcCLC1* and *BcCLC2*, qRT-PCR analyzed the relative expression levels of *BcCLCs* in mock and TuMV-infected NHCC. The results showed that the expression levels of both *BcCLC1* and *BcCLC2* were significantly increased in plants inoculated with TuMV (Figure 1). Thus, *BcCLC1* and *BcCLC2* may be responsive to TuMV infection.

### 2.2. Subcellular Localization of BcCLCs

**Figure 2:**
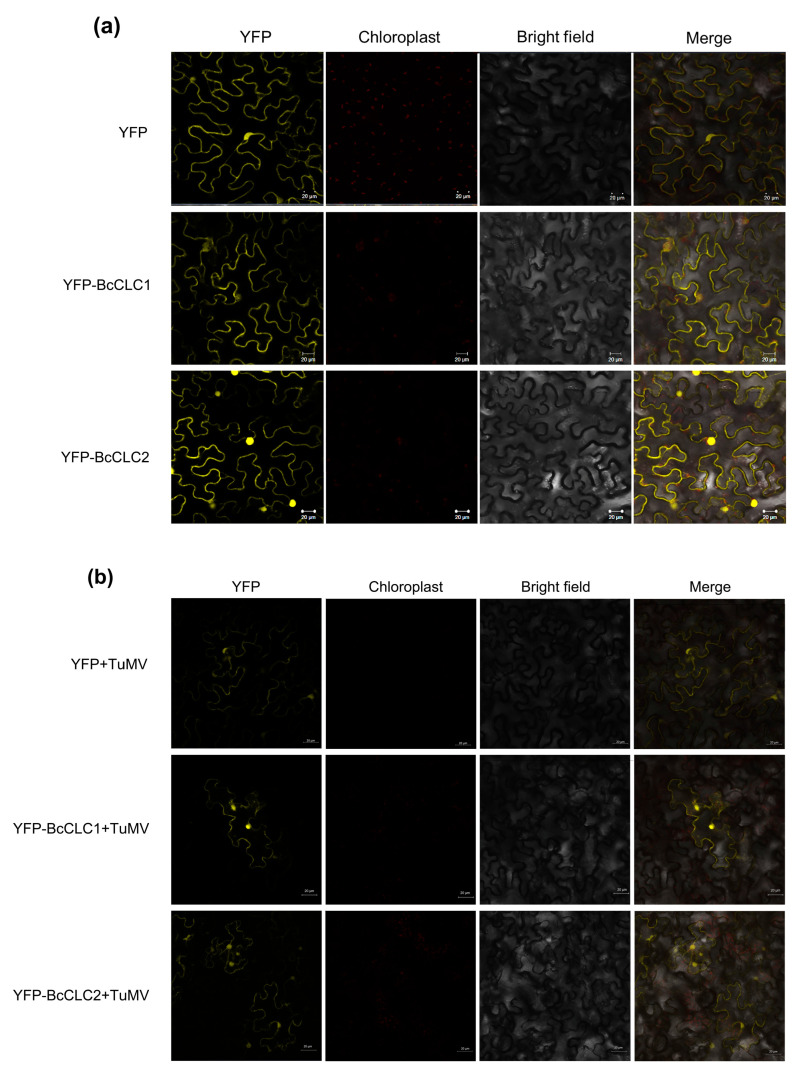
Subcellular localization of *BcCLCs*. (**a**) The subcellular localization of non-heading Chinese cabbage clathrin light chain proteins; (**b**) The subcellular localization of *BcCLC1* and *BcCLC2* infected by TuMV. Once inoculated for 72 h and observed using laser confocal microscopy, the yellow fluorescent signal was located in the yellow fluorescent protein (YFP) and bright fields, which were merged; chl: chloroplast fluorescence. The scale is 20 μm in size.

We generated a construct (35S: YFP-*BcCLC1*, 35S: YFP-*BcCLC2*) by fusing *BcCLC1* and *BcCLC2* copies into 35S: YFP, respectively, where 35S: YFP vector was used as a control. Using *Agrobacterium*-mediated transient transformation, 35S: YFP-*BcCLC1* and 35S: YFP-*BcCLC2* were transiently expressed in tobacco. Once infected for 72 h, we observed that signals from the control vector (35S: YFP) were located in the cytoplasm and nucleus. However, *BcCLC1* was mainly localized in the cell membrane and cytoplasm, with only a small amount entering the nucleus; *BcCLC2* was clearly distributed in the cell membrane, cytoplasm, and nucleus (Figure 2a). The two proteins are differentially localized, suggesting that the functions of the two clathrin light chain proteins diverged. TuMV was mixed with YFP, YFP-*BcCLC1*, and YFP-*BcCLC2* at OD600 concentrations adjusted to 0.6–0.8, and then injected into tobacco. Seventy-two hours later, using laser confocal scanning microscopy, fluorescence was observed (Figure 2b). The results showed that *BcCLC1* and *BcCLC2* were clearly distributed in the cell membrane and nucleus after TuMV infection.

### 2.3. Analysis of the Interaction between Clathrin Light Chain and the TuMV Protein in NHCC

This study used a BiFC assay and Y2H analysis to analyze the interactions between clathrin and 11 TuMV proteins.

**Figure 3:**
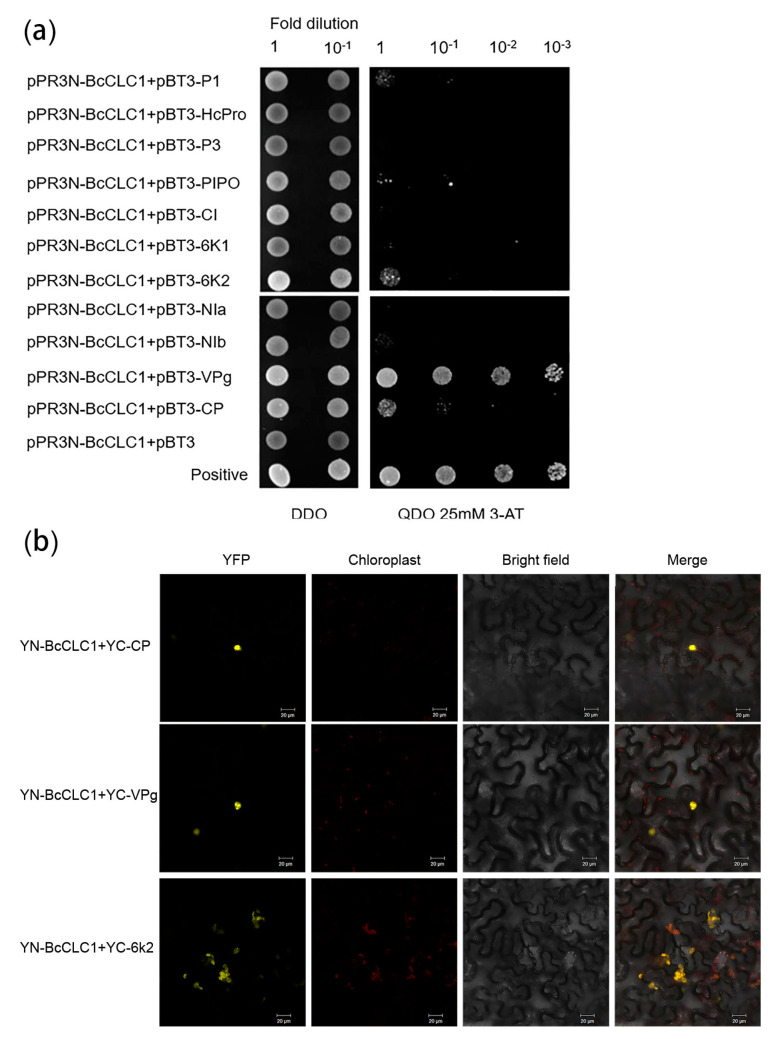
*BcCLC1* interacts with 6K2, CP, and VPg. (**a**) Y2H tests to detect the presence of interactions between *BcCLC1* and 11 TuMV proteins. The GAL4 activation domain was sequentially bound to *BcCLC1*, while 11 TuMV viral proteins were attached to the GAL4 binding domain. The plasmids were also diluted 10-fold and co-transferred into NMY51 yeast cells and coated onto SD/Trp-Leu medium after 3 days for positive screening. Positive control was pPR3N-At-DRP1A+pBT3-VPg, negative controls were pPR3N-*BcCLC1* and empty pBT3 co-transformed yeast cells; (**b**) BiFC assays to verify the interaction of *BcCLC1* with 6K2, VPg, and CP. Protein co-localization was observed after 72 h of tobacco infection. The pPR3N-*BcCLC1* was co-transformed with the YFP N-terminal fragment (YN). CP, VPg, and 6K2 were fused to YC. Bars, 20 μm.

The Y2H analysis was carried out to verify the interaction between TuMV proteins and *BcCLC1*. The binding of pPR3N-*BcCLC1* to 11 TuMV proteins occurred while transferring them into the NMY51 yeast strain. The results revealed that *BcCLC1* could interact with P1, PIPO, VPg, 6K2, and CP (Figure 3a). The strongest interaction was with VPg. It was subsequently verified by BiFC that *BcCLC1* could interact with 6K2, VPg, and CP. The location of the interactions with VPg and CP were mainly in the nucleus, and the location of the interaction with 6K2 was mainly in the outer chloroplast membrane (Figure 3b).

**Figure 4:**
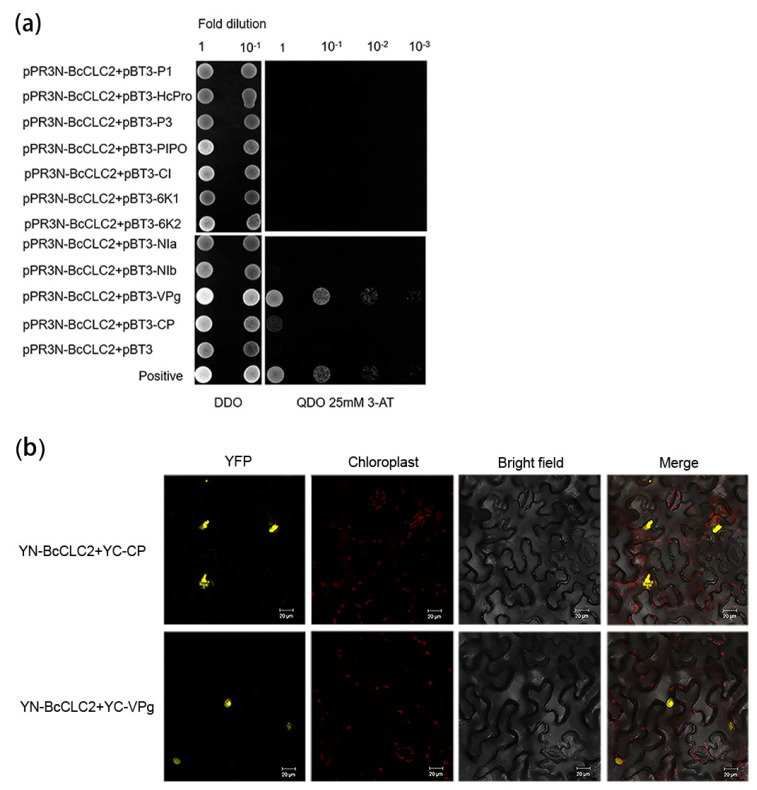
*BcCLC2* interacts with CP and VPg. (**a**) Identification of the interaction of *BcCLC2* with TuMV proteins through yeast two-hybrid; (**b**) Verification of the accuracy of Y2H by BiFC. Photographs were taken 72 h after *N. benthamiana* injection. Bars, 20 μm.

The same approach was taken for *BcCLC2* protein BiFC and Y2H tests. The interaction situation of the *BcCLC2* protein was verified by Y2H and it was found that it could interact with VPg and CP proteins (Figure 4a). In *N. benthamiana*, the interactions of *BcCLC2* with VPg and CP were again validated by BiFC (Figure 4b).

### 2.4. BcCLC1 Gene Silencing Inhibits the TuMV Infection of NHCC

**Figure 5:**
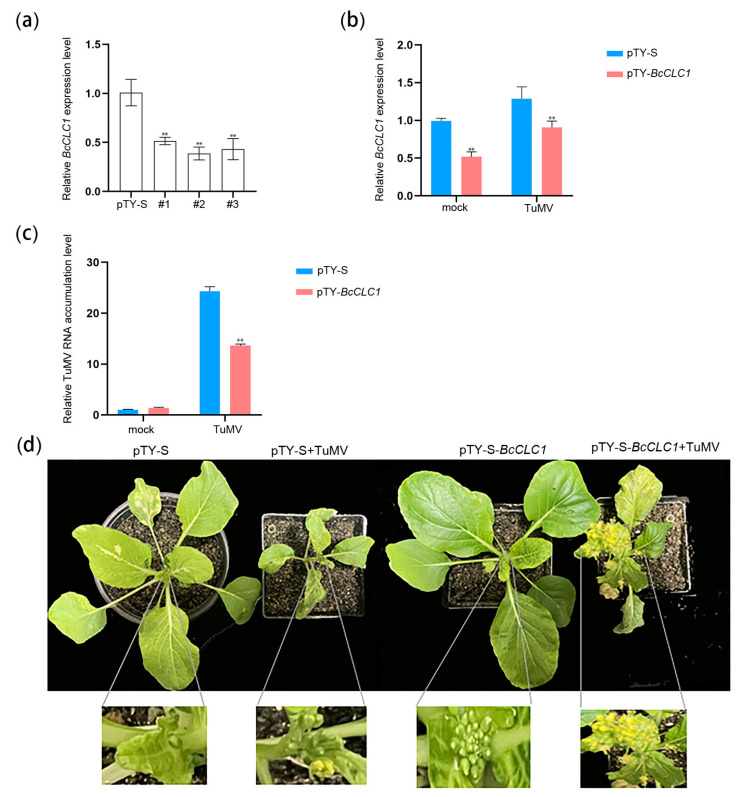
Silencing *BcCLC1* plants inhibited TuMV infection. (**a**) The relative expression level of *BcCLC1* in pTY-*BcCLC1* (#1, #2, #3) ‘49CX’ plants, with pTY-S as a control; (**b**) Verification of *BcCLC1* relative expression levels in the plants. Twenty days later, RNA was acquired from the diseased leaves. Errors are expressed as fold change ± SD of the control plants. The error of the three tests was shown by the error bars; (**c**) The relative expression level of TuMV genomic RNA in ‘49CX’; (**d**) Plants were inoculated with pTY-S (control), pTY-*BcCLC1* (*BcCLC1*-silenced) and infected with TuMV-GFP after the plants showed symptoms of the disease. Twenty days later plant phenotypes were taken; Differences between data were detected by Student’s *t*-test, **, *p* < 0.01.

To investigate the effect of VIGS on the expression of the *BcCLC1* gene in NHCC, a virus-induced gene silencing experiment was performed. The results of the trial revealed that VIGS reduced the relative expression level of the *BcCLC1* gene in NHCC. Following 2 weeks of bombardment of ‘49CX’ using the gene gun, we awaited the appearance of the obvious pTY virus phenomenon on the leaves of the silenced plants. RNA was obtained from leaves of the diseased plants, and the relative expression level of the silencing *BcCLC1* gene was identified by qRT-PCR using plants inoculated with pTY-S as a control (Figure 5a). The results indicated that the relative expression levels of *BcCLC1* were significantly lower in the three silenced strains compared to pTY-S, and showed highly significant differences.

We selected the leaves of the diseased plants and ground them into sap using a mortar and pestle with a buffer solution, and the ground pTY-*BcCLC1* (silencing *BcCLC1*) and pTY-S (control) sap were used to inoculate the ‘49CX’ plants. When the plants showed symptoms of disease, the plants were infected with the prepared sap of tobacco leaves containing TuMV -GFP. A qRT-PCR assay indicated that in the pTY-*BcCLC1* plants, the relative expression level of *BcCLC1* was reduced (Figure 5b). Figure 5c shows that there was less viral RNA accumulation by TuMV in the *BcCLC1*-silenced plants than in the control. According to Figure 5d, the *BcCLC1*-silenced plants had an enhanced resistance to TuMV compared with the controls, as demonstrated by the normal flower growth in *BcCLC1*-silenced plants, while the control plants were shorter and flowering was inhibited by TuMV infection. Above all, these results of these experiments revealed that *BcCLC1* silencing influenced the TuMV infection of NHCC and inhibited TuMV infection.

### 2.5. The Overexpression of BcCLC1 in Arabidopsis Promotes the TuMV Infection

**Figure 6:**
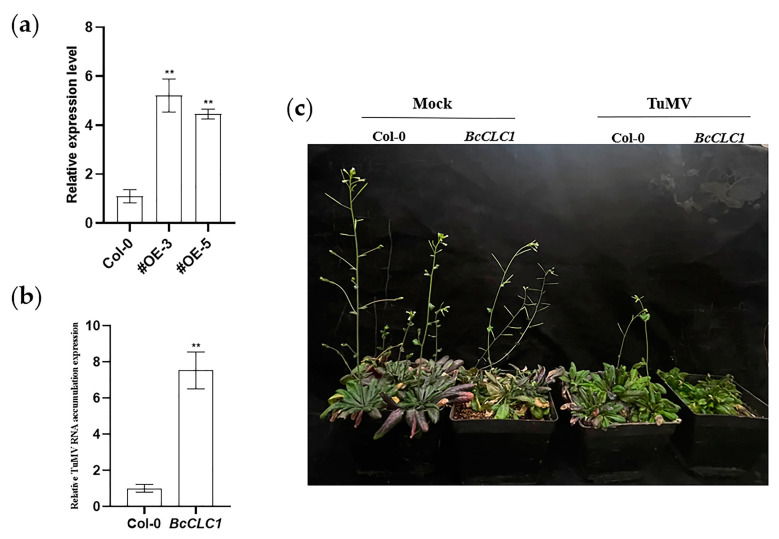
The overexpression of *BcCLC1* in *Arabidopsis* promotes the TuMV virus infection. (**a**) The relative expression level of the overexpression of the *BcCLC1* gene. #OE: overexpression of *Arabidopsis* plants (#OE-3, #OE-5); Col-0: WT, wild-type *Arabidopsis* plants; (**b**) The qRT-PCR examination the relative TuMV about RNA accumulation level of the Col-0 and overexpressed *BcCLC1* gene in *Arabidopsis* with TuMV infection at 15 d; (**c**) Phenotypes of Col-0 and the overexpression of *BcCLC1* in *Arabidopsis* after 15 d with TuMV inoculation; **, *p* < 0.01.

*Agrobacterium* containing the *BcCLC1* gene was transferred into *Arabidopsis thaliana* by the flower dipping method through an *Agrobacterium*-mediated transient expression method. Homozygous strains of the T_2_ generation were obtained through repeated selection, and the positive plants were numbered (#OE-3, #OE-5). The results of the qRT-PCR assay demonstrated that the expression level of the *BcCLC1* gene was significantly higher in the #OE-3 and #OE-5 strains than in WT (Figure 6a). To investigate the regulation of TuMV infection by *BcCLC1* in NHCC and the overexpression of *BcCLC1* in *Arabidopsis thaliana*, we used the *Agrobacterium*-mediated transient expression technique of flower dipping, and then we inoculated TuMV when screening for T_2_ generation positive plants. The qRT-PCR experiments indicated a significant increase in viral RNA accumulation by TuMV in the overexpression of the *BcCLC1* gene in *Arabidopsis* compared to the wild-type inoculated with TuMV (Figure 6b). Figure 6c shows that the overexpression of *BcCLC1* in *Arabidopsis thaliana* reduced the resistance of the plants to TuMV compared to the control. Therefore, we conclude that the overexpression of *BcCLC1* facilitated TuMV infection in NHCC.

### 2.6. BcCLC2 Silencing Inhibits TuMV Infection in NHCC

**Figure 7:**
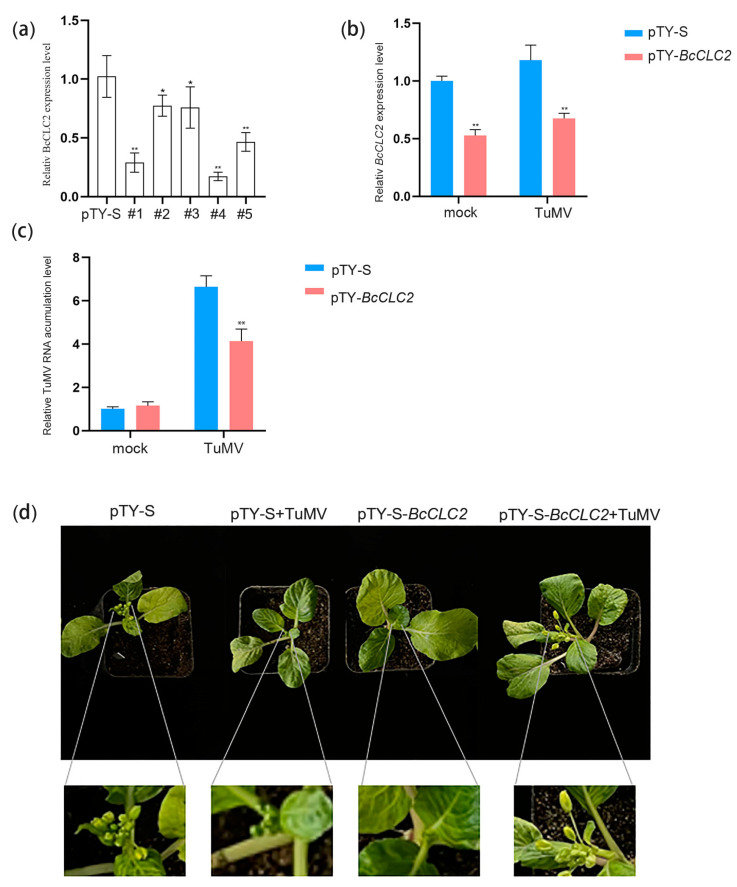
*BcCLC2* silencing suppressed TuMV infection in ‘49CX’. (**a**) The relative expression level of *BcCLC2* silencing in ‘49CX’ was significantly reduced. The expression level of *BcCLC2* in pTY-*BcCLC2* (#1, #2, #3, #4, #5) ‘49CX’ plants; (**b**) Fourteen days after the inoculation of ‘49CX’ with pTY-S and pTY-*BcCLC2*, followed by infection of plants with TuMV-GFP, and observation of the disease symptoms of the plants after twenty days; (**c**) The relative expression level of *BcCLC2* gene in ‘49CX’; (**d**) Relative accumulation level of TuMV genomic RNA in ‘49CX’; *, *p* < 0.05; **, *p* < 0.01.

The results demonstrated that VIGS reduced the expression of the *BcCLC2* gene in NHCC. RNA was extracted from diseased plants two weeks after the gene gun treatment with ‘49CX’, and the efficiency of *BcCLC2* gene silencing was determined by qRT-PCR, using pTY-S as a control (Figure 7a). The results indicated that the expression level of the *BCLC2* gene was significantly lower in the five silenced strains compared to pTY-S, with two plants showing significant differences compared to the control. The three plants showed highly significant differences.

As shown in Figure 7b, *BcCLC2*-silenced plants enhanced disease resistance against TuMV in ’49CX’ compared to pTY-S. The qRT-PCR results showed that *BcCLC2* was silenced in ‘49CX’, which was inoculated with pTY-*BcCLC2*, and Figure 7b shows that the relative expression level of the *BcCLC2* gene was significantly decreased in ‘49CX’, which was inoculated with pTY-*BcCLC2*. Compared to pTY-S, *BcCLC2*-silenced plants had reduced relative expression levels of TuMV genomic RNA (Figure 7c), a result that is also consistent with our phenotype (Figure 7d). In conclusion, the above experimental results revealed that *BcCLC2* silencing inhibited TuMV to infect NHCC.

### 2.7. Overexpression of BcCLC2 Positively Influences the Process of TuMV Infection in NHCC

**Figure 8:**
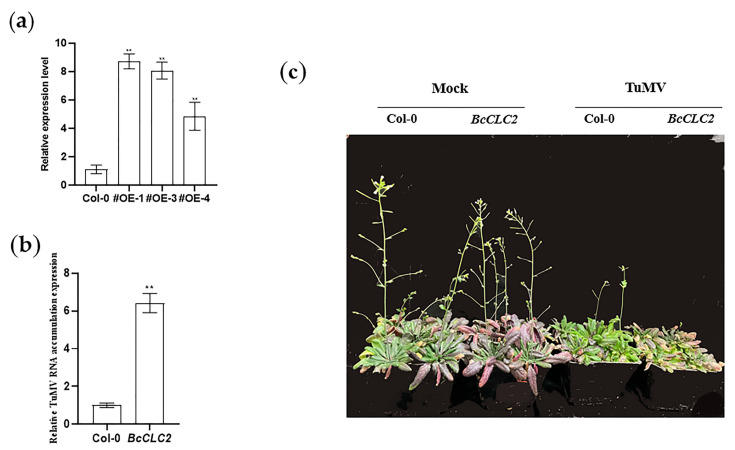
Overexpression of *BcCLC2* in *Arabidopsis* promotes TuMV virus infection. (**a**) Relative expression levels of *BcCLC2* overexpression; #OE: overexpression of *Arabidopsis* plants (#OE-1, #OE-3, #OE-4); (**b**) The qRT-PCR examination the relative TuMV about RNA accumulation level of the Col-0 and overexpressed *BcCLC2* gene in *Arabidopsis* with TuMV infection at 15 d; (**c**) Phenotypes of Col-0 and overexpressed *BcCLC2* in *Arabidopsis* after 15 d of TuMV inoculation; **, *p* < 0.01.

RNA was extracted from the overexpressed plants and wild-type plants. The qRT-PCR results showed that, based on the relative expression levels of *BcCLC2*, the #OE-3 and #OE-5 strains were identified as overexpressed *BcCLC2* plants (Figure 8a). The T_2_ generation of overexpressed pure *Arabidopsis thaliana* was inoculated with TuMV when the rosette leaves had grown but before the plants began flowering, and the Col-0 (wild type) was used as the control. Fifteen days after inoculation, *Arabidopsis* plants were photographed (Figure 8c), and the qRT-PCR assays indicated that the overexpression of *BcCLC2* in *Arabidopsis* up-regulated the accumulation of CP compared with the Col-0 (Figure 8b). According to Figure 8c, the overexpression of *BcCLC2* reduced the resistance of the plants to TuMV infection compared to the control. Therefore, we hypothesized that the overexpression of the *BcCLC2* gene promoted TuMV infection.

## 3. Discussion

Clathrin plays a key role in mediating the process of viral invasion in host cells. Several host factors associated with the endocytic pathway involved in viral infection have been screened and these host factors are associated with multiple aspects of viral infection. For example in animals, the tumor susceptibility gene *101* can interact with lattice proteins to mediate the endocytosis of the swine fever virus and regulate the viral replication by interacting with NS4B and NS5B viral proteins [23]. In plants, Wu et al. found that GmSDL5A, an initiator protein in the soybean CME pathway, promoted the infection of the *Soybean mosaic virus* (SMV). In *Arabidopsis*, the initiation protein AtDRP2 was also found to be recruited by a viral replication complex containing the 6K2 protein, demonstrating that initiation proteins in the plant CME pathway can facilitate *Potato virus Y* (PVY) infection [13,14]. To further test the mechanism of TuMV infection in NHCC, we performed VIGS and overexpression of *BcCLCs* assays. The results showed that *BcCLC1* and *BcCLC2* can interact with clathrin proteins to mediate the CME to promote the TuMV infection of non-heading Chinese cabbage.

Wang et al. found that, compared with the seedlings treated with *clc2-1* and *clc3-1* single mutants, the seedlings treated with *clc2-1 clc3-1* double mutants showed shorter axial roots and a longer hypocotyl. The plants treated with double mutants showed an increase in the degree of leaf curl and leaf length and a reduction in the number of trichomes at 3 weeks of age [16]. These results indicate that CME plays an important physiological role in signal transduction and polar auxin transport in plants. In this study, we silenced the *BcCLC1* and *BcCLC2* genes of non-heading Chinese cabbage using the VIGS technique, and the phenotypes of the silenced plants were similar to plants in previous studies (Figure 5d and Figure 7d). We concluded that the loss of clathrin resulted in auxin-related developmental defects affecting the growth and development of the plants.

Previous reports showed that *AtRIN13* was found to degrade CI by interacting with TuMV-encoded NIb and CI, thereby reducing its protein accumulation, which in turn led to the discovery that *AtRIN13* could degrade CI by way of the 20S proteasome for antiviral purposes [24]. Thus, we wanted to investigate whether such a mechanism also exists for clathrin light chains to regulate viral infections in NHCC. We designed Y2H and BiFC trials to verify the relationship between the 11 TuMV proteins and *BcCLC1* and *BcCLC2*. As we hypothesized, the results showed that *BcCLC1* can interact with 6K2, VPg, and CP. VPg is implicated in viral translation [25], in the long-distance movement in plant tissues [26], and in viral replication [27,28,29]. Also, 6K2 forms a polypeptide with VPg and enters the chloroplast as a site of viral replication through the vesicle structure produced by COPII [30]. Therefore, we hypothesize that *BcCLC1* may affect viral replication to regulate TuMV infection. BiFC showed that VPg and CP interacted with *BcCLC2*, which was verified using Y2H. CP protein glycosylation affects the stability of viral particles and increases the ability of viruses to infect their hosts [31]. CP is also involved in the movement of the virus between cells [32]. At the same time, it has been shown that CP is regulated by affecting the host, which in turn affects *Potato virus A* replication and virus accumulation [33]. Wang et al. found that loss of CLC2 and CLC3 affected CHC membrane binding and reduced internalization and intracellular transport of PM proteins [16]. We hypothesize that clathrin *BcCLC2* regulates TuMV to infect NHCC by regulating the virus transport pathway in cells by interacting with CP and VPg.

In conclusion, host factor genes *BcCLC1* and *BcCLC2* confer TuMV resistance in non-heading Chinese cabbage, and we speculate that *BcCLC1* regulates TuMV infection of NHCC by affecting virus replication, and *BcCLC2* positively regulates TuMV infection by regulating the virus transport pathways in cells. Nevertheless, the accuracy of our speculations will need to be investigated in the future. This experiment will provide a better understanding of the disease resistance genes in *Brassica*, explore the TuMV resistance genes in *Brassica*, and further understand the mechanism of action of clathrin in regulating TuMV infection of *Brassica*, and the implementation of this study will provide a new theoretical basis for improving disease resistance in *Brassica*.

## 4. Materials and Methods

### 4.1. Plant Materials and Growth Conditions

The seeds of NHCC cultivar ‘49CX’ were used for TuMV infection and the VIGS-induced gene silencing of *BcCLCs*. Following germination, ‘49CX’ seedlings were transferred to 72-hole plates containing cultivated soil (vermiculite: peat soil = [1:2]). The seedlings were placed under 16h 25 °C light/8 h 20 °C dark conditions for growth, and they remained in those conditions until the plants had grown 3–5 leaves. Robust and consistent growing materials were selected for TuMV infection and VIGS.

The plant used for TuMV inoculation and for the transient expression of the viral protein was the *Nicotiana benthamiana*, which was grown to 3–4 leaves. The cultural conditions were the same as ‘49CX’.

The Col-0 (wild-type) were first evenly sown on the surface of moist cultivation soil, vernalized at 4 °C for 2 days, cultured at 25 °C with moisture for about 10 days, and transpt lanted into 8 cm cultivation pots after they had grown two cotyledons. The transplanted seedlings were grown under 16h 25 °C light/8 h 20 °C dark conditions and, once they had grown 3–4 true leaves, were deemed appropriate for mutant identification or transgenesis after flowering.

### 4.2. Gene Cloning and Plasmid Construction

Specific primers were designed from the gene sequences of *BcCLCs* in the non-heading Chinese cabbage database (https://brassicadb.org/brad/, accessed on 12 May 2021) to clone the CDS. The primers are shown in Table S3. The coding sequences for *BcCLC1* and *BcCLC2* were amplified from NHCC cDNAs using PCR assays. This study used gateway technology to generate the clones reported. These were formed by recombining the cloned genes into pDONR221 using BP Clonase II according to Invitrogen’s instructions to form the introductory vector. Using LR Clonase II (Invitrogen), the target DNA was ligated to the target vector to form the expression vector and the exact sequence of the target gene was then confirmed by DNA sequencing. We performed comparison filtering of the local database using the last function of NCBI blast-2.9.0+ (https://ftp.ncbi.nlm.nih.gov/blast/executables/blast+/2.9.0/, accessed on 3 May 2021 ) with an e-value < 1.00E-100. We used Circoletto to visualize the blast results [17]. Clathrin from non-heading Chinese cabbage and *Arabidopsis* were compared using ClustalW [Multiple Sequence Alignment by CLUSTALW in MEGA-X (using default parameters)], which were accessed on 4 May 2021 in https://www.genome.jp/tools-bin/clustalw, the results were presented in Figures S1 and S2. Through ExPASy (https://web.expasy.org/protparam/, accessed on 3 May 2021) to predict theoretical isoelectric point and molecular weight of the protein, the results were presented in Tables S1 and S2.

### 4.3. Subcellular Localization and BIFC of BcCLCs

The entry vector was linearized with the restriction endonuclease MluI (NEB), and the expression vectors pEarlyGate101, pEarlyGate104, and YN were inserted for subcellular localization and BiFC, respectively.

The correctly sequenced expression vector was transferred into *Agrobacterium* GV3101, and a single colony was selected for overnight culture. The concentration of the subcellular localization carrier bacteria solution was adjusted to OD600 = 0.3, and the tobacco leaves were injected. YN-*BcCLC1* and YN-*BcCLC2* were mixed with YC-P1, YC-HC-Pro, YC-P3, YC-PIPO, YC-6K1, YC-CI, YC-6K2, YC-NIa, YC-VPg, YC-Nib, and YC-CP in equal amounts, respectively. The final concentration of each component OD600 = 0.3 was injected into tobacco leaves. The localization and interaction were observed under the laser confocal microscope 72 h after injection.

### 4.4. Y2H Analysis

The full-length CDS of *BcCLC1* and *BcCLC2* were connected to yeast two-hybrid vector pPR3-N, respectively. The 11 TuMV genes were inserted into the pBT3-STE vector, and the primers are shown in Table S1. We predicted the protein interactions by observing the yeast growth on media lacking different nutrients. Their interactions were tested using the same method.

### 4.5. TuMV Inoculation

A solution of *Agrobacterium tumefaciens* containing TuMV-GFP was adjusted to OD600 = 0.3–0.4 and left at room temperature for 3–4 h to infect *N. benthamiana* grown for about 6 weeks. Inoculation was carried out by friction inoculation. Diseased tobacco leaves were ground in a mortar and pestle with a phosphate buffer (0.05 mol/L) to form a sap. The sap was filtered through gauze and then infected with ‘49CX’ and *Arabidopsis* using the friction method.

### 4.6. Virus-Induced Gene Silencing Experiment

Silent ‘49CX’ plants were obtained using the VIGS technique as previously reported [34]. 40 bp base sequences were selected from the *BcCLC1* and *BcCLC2* gene sequences, respectively, which formed an 80 bp fragment with reverse complementary sequences (Table S4), and the plasmid synthesis was performed at GeneScript Company (Nanjing, China). The pTY-*BcCLC1* and pTY-*BcCLC2* glycoconjugates synthesized by the company were expanded and the plasmids were extracted using the plasmid extraction kit from Beijing Tiangen for use in the subsequent experiments. Thereafter, gold powder was used to coat the pTY and pTY-*BcCLCs* plasmids, and they were then subjected to gene gun-mediated transformation (PDS-1000/He, Bio-Rad, Hercules, CA, USA) and implanted into plants grown to two true leaves, with six plants bombarded per plasmid at a time and three replicates. Three replications were performed. Fourteen days later, leaf disease symptoms became apparent and leaves with disease phenotypes were sampled to identify the relative expression level of the silenced genes by qRT-PCR silencing efficiency. The leaves of plants with high gene silencing efficiency and pTY-S were recovered, ground in PBS buffer and stored at −70 °C.

### 4.7. Screening for the Overexpression of BcCLCs in Arabidopsis

To obtain the transgenic overexpression of *BcCLC1* and *BcCLC2*, *Arabidopsis thaliana* was transformed with 35S:pEarleyGate104-*BcCLC1*-GFP and 35S:pEarleyGate104-*BcCLC2*-GFP using the flower dipping method, respectively. Transgenic *Arabidopsis* seeds were screened using 1/2 MS solid medium with 16 mg/L Timetin and 50 mg/L Basta. Seeds obtained from transgenic *Arabidopsis* were referred to as T_1_ generation seeds. They were screened using the same method as the T_2_ generation seeds, and then they were selected for the inoculation of TuMV-GFP on T_2_ generation *Arabidopsis* for the subsequent qPCR analysis and phenotypic observation.

### 4.8. RNA Extraction and qRT-PCR

RNA was extracted from the leaves of diseased plants 14 days after TuMV infection. Meanwhile, a Pure Plant Total RNA Isolation Kit was provided by TIANGEN Biochemical Technology (Beijing, China). The cDNA was then obtained in two steps using the *Evo M-MLV* RT Kit with gDNA Clean for qPCR (AG, Hunan, China). Then qRT-PCR was performed using 2 × Hieff^®^ qPCR SYBR Green Master Mix provided by YEASEN Biotechnology (Shanghai, China) to identify the relative expression levels of the genes. *BcActin* was used as a qRT-PCR internal reference gene. The qRT-PCR tests were used to determine the relative expression levels of genes or viruses. The primers used in this study are listed in Table S3. The data were calculated using the 2^−∆∆ CT^ method [35].

### 4.9. Statistical Analysis

The trials in this research were biologically replicated at least three times. The error bars were used to indicate the sample standard deviation, and the differences between data were identified using Student’s t-test. The differences between data were identified using ANOVA-LSD in the Figure 5a, Figure 6a, Figure 7a and Figure 8a. *p* < 0.01 indicates a highly significant difference. *p* < 0.05 indicates significant difference. Since there is no mutant library in non-heading Chinese cabbage, in order to study the response of plants to TuMV when knocking out clathrin, this study screened and detected the mutant homologous gene of *BcCLCs* in *Arabidopsis*, the result was presented in Figure S3.

## Data Availability

All the data are in the manuscript.

## Supplementary Materials

The following supporting information can be downloaded at: https://www.mdpi.com/article/10.3390/plants12122269/s1, Figure S1: Amino acid sequence alignment of BcCLC1 and AtCLC1 and structural domain analysis of BcCLC1; Figure S2: Amino acid sequence alignment of BcCLC2 and AtCLC2 and structural domain analysis of BcCLC2; Figure S3: The identification of AtCLCs mutants. Table S1: The correspondence between NHCC and Chinese cabbage clathrin proteins; Table S2: The correspondence between NHCC and Chinese cabbage; Table S3: Primers were used in this study; Table S4: Sequences for construction of the VIGS vector.
