# Supplementary material for: Host Factors Genes BcCLC1 and BcCLC2 Confer Turnip Mosaic Virus Resistance in Non-Heading Chinese Cabbage (Brassica campestris ssp. chinensis)
Source: Plants (Basel). 2023 Jun 10;12(12):2269. doi: 10.3390/plants12122269 (PMC10304732; doi:10.3390/plants12122269)
Supplement: Supplementary file 1 [file plants-12-02269-s001.zip › plants-2309079-supplementary.pdf]

(a)

(b)

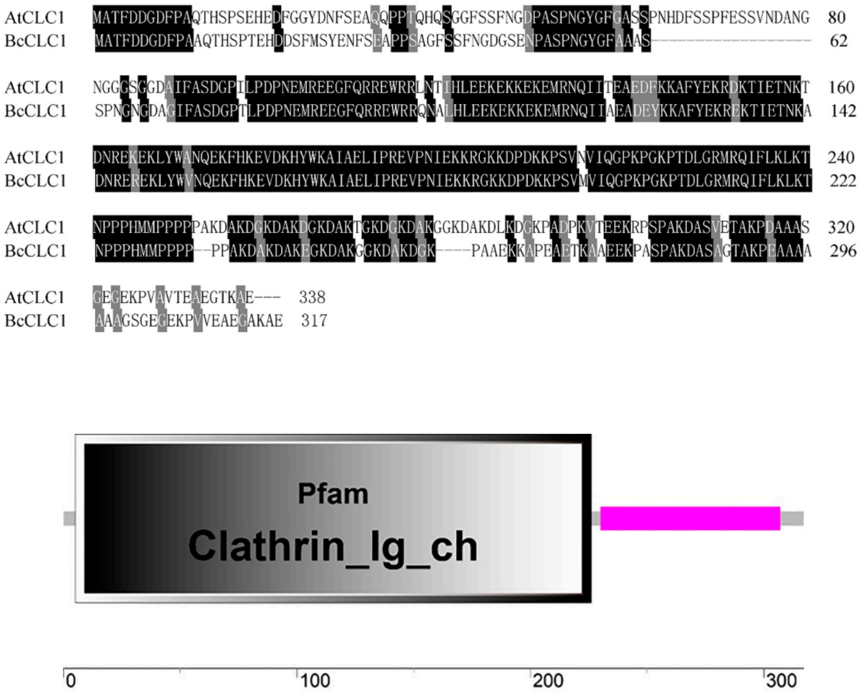

Figure S1. Amino acid sequence alignment of BcCLC1 and AtCLC1 and structural domain analysis of BcCLC1. (**a**)Comparative amino acid sequences of BcCLC1 and AtCLC1. The amino acid sequence identity between BcCLC1 and AtCLC1 was 63.05% and the similarity rate was 70.67%. Black background indicates perfect agreement, grey background indicates conserved regions; (**b**) SMART structural domain analysis of BcCLC1, the grey background indicates that the grey line segment is the full length of the amino acid and the red rectangle indicates the low complexity region, with the ruler in the lower part.

(**a**)

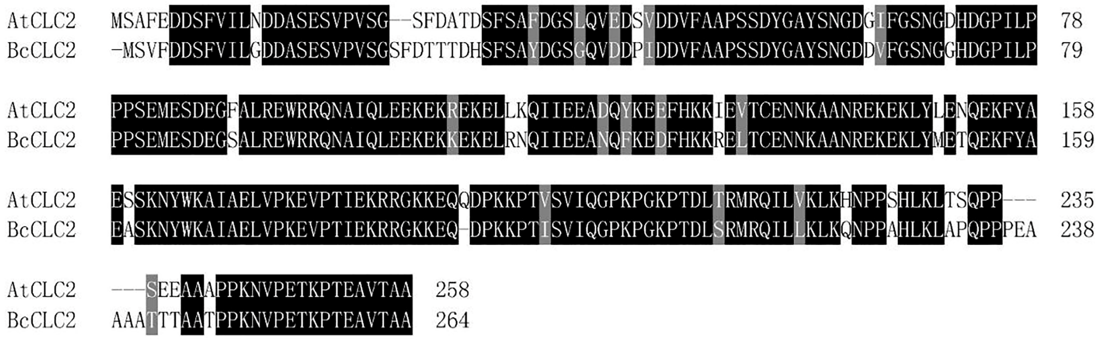

(**b**)

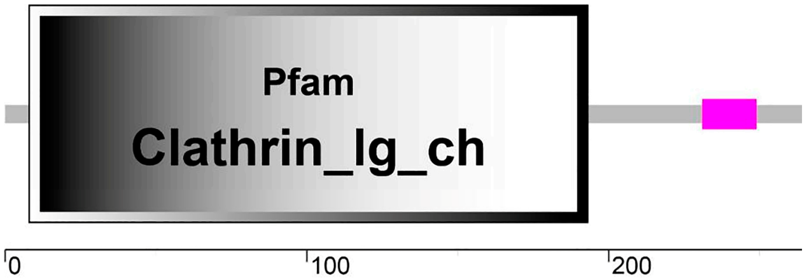

Figure S2. Amino acid sequence alignment of BcCLC2 and AtCLC2 and structural domain analysis of BcCLC2. (**a**)Comparative amino acid sequences of BcCLC2 and AtCLC2. The amino acid sequence identity between BcCLC2 and AtCLC2 was 80.45% and the similarity rate was 85.71%. Black background indicates perfect agreement, grey background indicates conserved regions; (**b**) SMART structural domain analysis of BcCLC2, the grey background indicates that the grey line segment is the full length of the amino acid and the red rectangle indicates the low complexity region, with the ruler in the lower part.

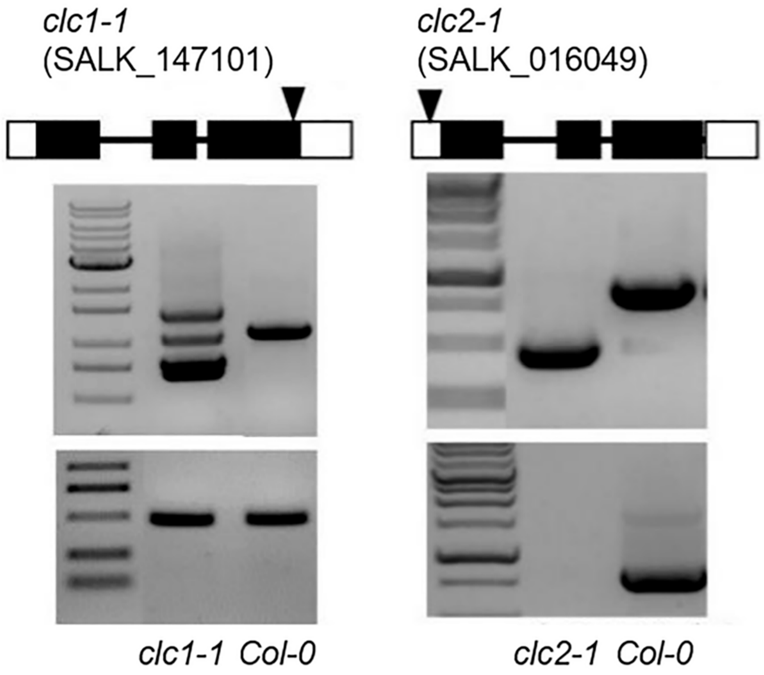

Figure S3. The identification of AtCLCs mutants. White rectangles denote 5'UTR or 3'UTR, black rectangles represent exons, black line segments represent introns and black arrows represent T-DNA insertion sites.

Table S1. The correspondence between NHCC and Chinese cabbage Clathrin proteins

Num	NHCC_genes	At_genes	e-value	chr	Strand	pos_start	pos_end	length	Protein length	MV	PI
1	BraC09g064010.1	*AtCLC1*	2.00E-132	A09	+	59541847	59543654	954	318	34.8kDa	5.74
2	BraC03g021570.1	*AtCLC2*	7.00E-142	A03	+	10760457	10762086	795	265	29.2kDa	5.10
3	BraC05g006400.1		2.00E-131	A05	-	3593438	3594647	741	247	27.3kDa	5.44
4	BraC04g030120.1		1.00E-114	A04	+	21291217	21292532	699	233	25.9kDa	5.29

chr: chromosome; MV: molecular weight; PI: isoelectric point.

Table S2. The correspondence between NHCC and Chinese cabbage

Query Item	String Id	Identity	Bit score	Preferred Name	Annotation
BraC09g064010.1	3711.Bra031138.1-P	99.6	387.1	Bra031138.1-P	Clathrin light chain
BraC03g021570.1	3711.Bra000163.1-P	100	444.5	Bra000163.1-P	Clathrin light chain

Table S3. Primers were used in this study

Primer	Sequence(5′-3′)
**For gene cloning**	
gw-*BcCLC1*-F	ggggacaactttgtacaaaaaagttggcatggcGACTTTCGATGACGGA
gw-*BcCLC1*-R	ggggacaactttgtacaagaaagttgggcaCTCTGCCTTGGCTCCCTCAG
gw-*BcCLC2.1*-F	ggggacaactttgtacaaaaaagttggcATGTCTGTCTTTGACGATTCCTTCG
gw-*BcCLC2.1*-R	ggggacaactttgtacaagaaagttgggcaAGCAGCAGTAACTGCCTCGG
**For Y2H**	

PPR3N-BcCLC1-F	gagtggccattacggccATGGCGACTTTCGATGACGG
PPR3N-BcCLC1-R	gagaggccgaggcggccgCTCTGCCTTGGCTCCCTCAG
PPR3N-BcCLC2.1-F	gagtggccattacggccATGTCTGTCTTTGACGATTCCTTCG
PPR3N-BcCLC2.1-R	gagaggccgaggcggccgAGCAGCAGTAACTGCCTCGG
**For qRT-PCR**	
BcACTIN-qPCR-F	GTTGCTATCCAGGCTGTTCT
BcACTIN-qPCR-R	AGCGTGAGGAAGAGCATAAC
AtActin2-qPCR-F	CACCACAACAGCAGAGCGGGA
AtActin2-qPCR-R	TCCCACAAACGAGGGCTGGA
BcCLC1-qPCR-F	CCGTCAAAACGCACTTCACC
BcCLC1-qPCR-R	GGCCTTCTTGTATTCGTCAGC
BcCLC2-qPCR-F	GTCTCAGGCTCATTCGACACC
BcCLC2-qPCR-R	TCCGTTCGAGTAAGCTCCGTA
TuMV-CP-qPCR-F	TGGCTGATTACGAACTGACG
TuMV-CP-qPCR-R	CTGCCTAAATGTGGGTTTGG
**For mutant identification**	
LBb1.3	ATTTTGCCGATTTCGGAAC
SALK_147101-LP	CATGCCCCACTCTCTCTAATG
SALK_147101-RP	GCATTACTGGAAAGCAA TTGC
SALK_016049-LP	GCTCAATGATTGTGCCAATTC
SALK_016049-RP	GCCATAGCACGAAATCAGATC
CS100219-LP	GACGGAGGAAACTTCACG GCTTAC
CS100219-RP	CACTCACACTCACATTCGGGTCAG

Table S4. Sequences for constrction of the VIGS vector

Gene Name	Sequence（5'-3'）
BcCLC1	GTGAGTGGCGCCGTCAAAACGCACTTCACCTCGAGGAGAATTCTCCT CGAGGTGAAGTGCGTTTTGACGGCGCCACTCAC
BcCLC2	ATGACCCCATCGACGATGTTTTCGCAGCGCCGTCCTCCGATCGGAGG ACGGCGCTGCGAAAACATCGTCGATGGGGTCAT
